# The effect of educational support intervention including peer groups for infant care on the growth rates of infants, breastfeeding self-efficacy and quality of life of their mothers in Iran: study protocol

**DOI:** 10.1186/s12978-022-01523-6

**Published:** 2022-12-01

**Authors:** Forogh Okhovat, Shirin Okhovat, Zohreh Karimiankakolaki, Nooshin Yoshani

**Affiliations:** 1 Nursing and Midwifery Care Research Center, School of Nursing and Midwifery, Iran University of Medical Science, Tehran, Iran; 2 Student Research Committee, School of Nursing and Midwifery, Isfahan University of Medical Sciences, Isfahan, Iran; 3 Department of Health, Shahrekord Branch, Islamic Azad University, Shahrekord, Iran; 4 Department of Health Education and Health Promotion, Social Determinants of Health Research Center, School of Public Health, Shahid Sadoughi University of Medical Sciences, Yazd, Iran

**Keywords:** Infants, Breastfeeding, Quality of life, Mothers, Breastfeeding self-efficacy, Peer groups

## Abstract

**Background:**

Mothers' knowledge about the infant's problems and will provide the necessary care can increase the mother's confidence in providing care for her infant and eliminate misconceptions in primiparous mothers. The present study will be conducted to evaluate the effect of educational support intervention including peer groups for infant care on the growth rates of infants, breastfeeding self-efficacy and quality of life of their mothers in Iran.

**Methods:**

This quasi-experimental study is an educational support intervention protocol for infant care which will be conducted in two phases. The educational support program will be designed in the first phase. The program includes educational sessions on breastfeeding, growth and development of infants and care for children under 1 year of age in a virtual group where a physician, a nurse, a midwife and a healthcare provider are also present. Using the opinions of the experts, the peer group will discuss various points and the research team will finalize the program based on priorities. The second phase of the educational intervention will be conducted experimentally as a pretest–posttest design for the intervention and control groups.

**Discussion:**

The present study will provide useful protocol about the effect of educational support intervention for infant care and the sharing of peer group experiences on infants' growth rates, breastfeeding self-efficacy, and quality of life of their mothers. The current educational package not only combines virtual education and peer experiences in strengthening the education of mothers, but also it can improve mothers' physical and mental health and reduce medical costs by using telephone follow-up in supporting of the mothers of infants.

*Trial registration:* Registration of this randomized control trial has been completed with the Iranian Registry of Clinical Trials, IRCT20210913052457N1, registered 9/10/2021, https://www.irct.ir/trial/59093.

## Plain language summary

Mothers' knowledge about the infant's problems and provide the necessary care can increase the mother's confidence in providing care for her infant and eliminate misconceptions in primiparous mothers. This quasi-experimental study is an educational support intervention protocol for infant care that will be conducted in two phases. The educational support program will be designed in the first phase. The program includes educational sessions on breastfeeding, growth and development of infants and cares for children under one year of age in a virtual group where a physician, a nurse, a midwife and a healthcare provider are also present. Using the opinions of the experts, the peer group will discuss various points and the research team will finalize the program based on priorities. The second phase of the educational intervention will be conducted experimentally as a pretest–posttest design for the intervention and control groups. The present study will be conducted to evaluate the effect of educational support intervention including peer groups for infant care on the growth rates of infants, breastfeeding self-efficacy and quality of life of their mothers in Iran.

## Background

According to global nutritional goals and policies, by 2025, the rate of exclusive breastfeeding at 6 months should reach at least 50% [1]. The amount of exclusive breastfeeding varies in countries and even in different regions of the same country, such that exclusive breastfeeding has been reported in Japan is 21% [2], India 7.8% [3], Canada 6% [4], and Ghana 6.51% [5]. In Iran, according to reports, exclusive breastfeeding is 22.7% in urban areas, and 24% in rural areas, which is significantly different from WHO goals [6]. As one of the most sensitive stages of life, infancy is a period that needs to be correctly known, and precisely cared. The maintenance and promotion of children's health as a vulnerable group has a special place in health services, improving the level of knowledge and removing the false beliefs of mothers in caring for infants will lead to an increase in mother's self-confidence [7]. 

According to many studies, paying attention to care in the first years of an infant's life is of particular significance [8]. Accordingly, to prevent underweight, interventional measures should be taken before the age of two [9]. The mother's satisfaction with the quality of the baby's nutrition is a predictive variable of the breastfeeding self-efficacy score [10], according to studies, the mean score of breastfeeding self-efficacy in a study in Iran was 47.01, and in another study, this rate was 48.1 [11, 12]. The responsibility of infant care since birth has been to a large extent on the shoulder of mothers. As such, raising the awareness of mothers, their empowerment and improving their quality of life are the main objectives of the healthcare system. Mothers may experience various physical and mental complications during childbirth and the postpartum period, which can affect the natural process, and quality of their life [9, 13]. Quality of life is a feeling of well-being that arises from satisfaction or dissatisfaction with various life aspects that are of great importance to the individual; it includes the areas of health, occupation, socio-economic, psychology and family, and is a significant healthcare measurement criterion [14]. Chen et al.'s study showed that the quality of life of mothers who used different breastfeeding patterns and had a breastfeeding duration of 6 months or more had higher quality of life, this rate is also reported to be higher in mothers who exclusively breastfeed for up to 6 months [15]. Given the fact that most postpartum problems related to mother and infant are solved easily and through education, paying attention to the method, content and modality of these educations seems to be important [16]. The creation of positive self-efficacy, especially self-efficacy in breastfeeding, seems to be a prerequisite for mothers' empowerment [17]. The continuation of breastfeeding is easier in mothers with high self-efficacy in breastfeeding. According to researchers, mothers with more successful breastfeeding will more successfully raise their children and will have a stronger sense of accepting their motherly role. Bandura believes that one's level of empowerment and self-efficacy can be improved by adopting appropriate educational strategies and interventions in acquiring the necessary skills, and knowledge [18].

Today, e-learning is used by patients as the main part of healthcare. E-learning has been introduced by the World Health Organization as an appropriate way of communicating with clients [19, 20]. While virtual space and direct education through telemedicine have an overt role in the health system, the role of covert education through other clients with similar health problems cannot be ignored. In education through the experiences of peers, simple and safe learning is created based on the similar features of the members of a group; in this method, clients share their healthcare-related experiences [21]. Additionally, peer groups are better able to encourage their peers to choose appropriate health behaviors; they are also able to share common strengths, weaknesses, and experiences [22]. The need of mothers for breastfeeding-related scientific information and caring for their infants in encountering misconceptions reveals the need for education on the key points [23]. The positive role of educational programs in improving breastfeeding outcomes has been confirmed in various studies. However, no study has hitherto investigated the effect of a virtual educational-supportive program of infant care by sharing peer experiences on the growth indicators of infants, breastfeeding self-efficacy and quality of life of mothers. Thus, the present study will be conducted to bridge this gap.

## Aim

This study seeks to design and implement an educational support program for infant care by sharing the experiences of peers, which will be able to affect the indicators of infant growth, breastfeeding self-efficacy, and quality of life of mothers.

## Research hypotheses

The hypothesis is that after the intervention, the mean scores of infant growth indicators, mothers' breastfeeding self-efficacy, and mothers' quality of life will increase compared to the control group.

## Methods

This clinical trial study is an educational support intervention protocol for infant care which will be conducted in two phases. The educational support program will be designed in the first phase. This program will be designed based on the review of previous studies and considering the opinions of the panel of experts in health education and promotion, pediatric nursing, and mother-infant health. The program includes educational sessions on breastfeeding, growth and development of infants and cares for children under one year of age in a virtual group where a physician, a nurse, a midwife and a healthcare provider are also present. Using the opinions of the experts, the peer group will discuss various points and the research team will finalize the program based on priorities. Reminder text messages will be sent to the participants to remind them of the educational program. The second phase of the educational intervention will be conducted experimentally as a pretest–posttest design for the intervention and control groups.

## Study site and population

This clinical trial study will be conducted at the health centers in Yazd, Iran. Mother-infant pairs living at Yazd will be recruited from three health centers in the upper, lower and center of the city will be included in the study and will receive primary health care related to their infants according to the intervention program (virtual group and expert and peer training). In this study, the control group will have the routine services of health centers. These services include visiting the health center in person and checking the growth and development of the infant and receiving care information from the health team members.

## Phase I: designing an educational-supportive program

The design of the intervention and the identification of the target audience in this phase will be based on the review of the texts and similar studies as well as the opinions of experts. The educational support program will include virtual training sessions on breastfeeding, growth and development of infants and care for children under one year of age in a WhatsApp group where a physician, a nurse, a midwife and a healthcare provider are among the members. Moreover, the experiences of the peers will be used through discussions and the exchange of information. The studied population will be mother-infant pairs less than one year of age referring to Yazd health centers. The researcher will receive the mothers' phone numbers through the Sib system and will call to the mothers, and hold a face-to-face meeting at the health center.

### Virtual educational support sessions

All mothers of the intervention group will join the WhatsApp group, which is managed by the researchers. Additionally, other members of the virtual group include a midwife, a nurse, a healthcare provider and a family physician for answering the specialized questions of the mothers. Accordingly, about their questions, the mothers will have access to nurses, physicians and midwives.

In this group, 2 days a week, an educational program will be provided by the researcher in the form of educational packages related to breastfeeding, growth and development, vaccination, health and nutrition and other care for children under one year of age for two months. The educational content has been shown in Table 1. These packages will be sent to mothers based on the latest references from pediatric nursing books approved by the Ministry of Health and the family physician. Two days after sending the educational materials to the mothers, the researcher (pediatric nurse) will provide the mothers with additional explanations about the educational content for 40 min and the mothers will ask their questions. In this study, the control group will confirm the demographic information form and the consent form, and they will complete the quality of life and breastfeeding self-efficacy questionnaires before and after the intervention, and during the intervention, they will benefit from the routine services of health centers. These services include visiting the health center in person and checking the growth and development of the infant and receiving care information from the health team members.

**Table 1:** Educational content

Educational program	Educational content	How to provide education	Audience
Educational package			
Newborn	Breastfeeding methods (time and duration of breastfeeding); milking and storing breast milk; bathing the infant; vaccination and aftercare; umbilical cord care; change of clothes; burping; methods of preventing sudden infant death syndrome; how to use supplements; Thyroid check-up (hypothyroidism training); education of danger and safety symptoms and the need for seeing a physician; and audiometry	The educational program will be offered two days a week and for two months in the form of an educational package which will be sent to the virtual group	Mothers of infants
One month to 1 year old	Optometry; complementary nutrition; toilet training; infant safety; food allergies; vaccinations and aftercare; weaning education; oral health of the infant; development of social skills as well as delicate and coarse movements		
Question and answer	Questions about the educational content and problems of the mothers and the response of the experts to these questions in the group	Necessary additional explanations about the educational content and questions asked by the mothers in the virtual group	Mothers of infants
Peer experiences	Sharing the experiences of mothers about the growth and development of their infants in a virtual group and under the supervision of experts	Sharing the experiences of mothers in a virtual group	Mothers of infants
Education follow-up	Message for reminding of educational content together with verbal encouragement	Sending reminder messages at the end of each week	

### Peer group experiences

In addition to virtual sessions, the mothers of this group will be able to share their experiences about the growth and development of their infants in this space for three months. At the beginning of the study, the mothers will be informed that the prescription of chemical, herbal, and traditional medicines is against the rules of the research plan and in case of violating these rules, they will be removed from the group. Sharing the experiences of mothers is limited to the issues of growth and development of a healthy child. The researcher and family physician will control all the messages of the intervention group members and prevent the mothers from confusion by providing them with correct guidance. Moreover, in case of illness and any clinical problems for the infant, it is recommended that mothers see a specialist in person.

### Follow-up and sustained education

For follow-up and sustained education, consolidate the educational content, at the end of each week, a summary of the taught content will be sent to mothers as a reminder. Also, to ensure the effectiveness of the training, at the end of each month, mothers will evaluate by answering questions about the content of the training materials.

### Expert’s team

The content and visual validity of the educational program will be measured, and the comments received from the panel of experts. The panel of experts will be included 4 professionals from the health education and health promotion field, 3 professionals from reproductive health and 2 experts from the field of pediatric nurses and a doctor.

## Phase II: implementation of educational intervention

**Fig. 1:**
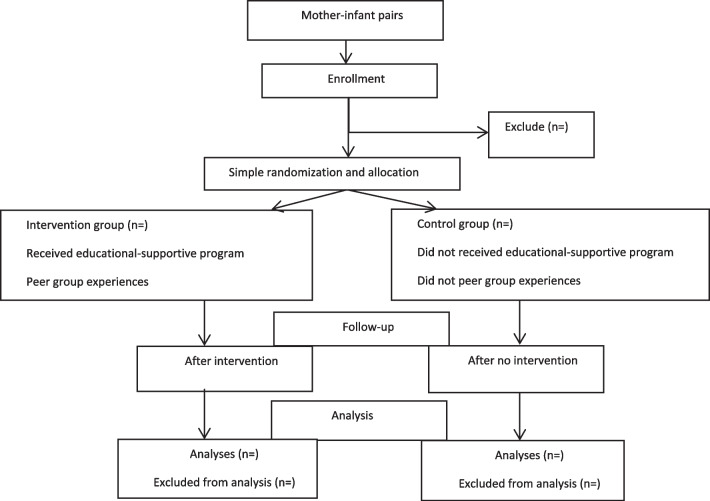
Flow chart of study design and population

In this step a randomized controlled clinical trial will be design to assess the efficacy of the prepared protocol. Participants will randomly be allocated into two groups. In the experimental group we will implement our designed protocol, and the control group will not receive any intervention, but they are evaluated as the same as the experimental group. For ethical issues all educational material will offer at the end of the study (Fig. 1).

### Study sample

This interventional randomized study will be conducted on mothers of infants who have been receiving maternity care at health centers in Yazd, Iran. According to the study of Araban et al. [24] and considering the level of significance (α = 0.05) and test power (β = 0.8) as well as the standard deviation of breastfeeding self-efficacy score which is 4.22 and 4.88 in case and control groups respectively, and in order to achieve a significant difference of at least 2.5 points in the mean score of breastfeeding self-efficacy in the intervention and control groups, and taking into account 10% of probable drop, the sample size is considered to be 57 subjects in each group using the following formula.

$$n = \frac{{\left( {Z_{{1 - \frac{\alpha }{2}}} + Z_{1 - \beta } \left( {\sigma_{1}^{2} + \sigma_{2}^{2} } \right)} \right)}}{{d^{2} }}$$

### Sampling method

A random sampling method will apply to select the participants. The sampling method will be random and the participants will be in two groups’ intervention and control. The selection of participants to enter the study will be based on a table of random numbers, and to randomize the number of people in the control and intervention groups, cards will be prepared and placed in an envelope, then the participant will be asked to choose a card and based on the lottery will be placed in the control or intervention group.

### Inclusion criteria

Inclusion criteria include willingness to participate in the study, primiparous mothers, having an under one-year-old infant, physical health of the child and absence of chronic diseases and chromosomal-genetic defects, no underlying disease, access to mobile phones for connecting to virtual space, ability to use internet and media literacy. Iranian nationality, consent to participate in the study, ability to understand questions or literacy for reading and writing, and not participating in any other clinical trials at the same time.

### Exclusion criteria

The diagnosis of chronic illness of the baby, the death of the baby, and the unwillingness of the mother to continue cooperation are the exclusion criteria.

### Data collection method

#### Quality of life questionnaire

The SF-36 Quality of Life Questionnaire is a general questionnaire for assessing the quality of life of individuals; it assesses the quality of life of individuals in 8 domains of physical function, limitations caused by physical problems, social functioning, physical pain, and mental health, limitations caused by mental problems, vitality and general health. The score of each domain is calculated independently and ranges from zero (the worst condition) to 100 (the best condition). Questions of the mentioned dimensions are as follows: physical function (questions 3 to 12); limitation caused by physical problems (13 to 16); social functioning (2, 20 and 32), physical pain (21 and 22), mental health (24 to 28 and 30); limitations caused by mental health problems (17 to 19), vitality (23, 27, 29, 31) and general health (1, 33 to 36). The reliability of this questionnaire has been calculated to be 0.89 and 0.92 by retest and Cronbach's alpha respectively [14].

#### Breastfeeding self-efficacy scale

The breastfeeding self-efficacy scale is derived from the short form of BSES-SF Dennis. This 14-item scale is designed based on a 5-point Likert scale; so that, I am not sure at all is given score 1, and scores 2 to 5 are given to a bit sure, relatively sure, I am sure, and I am quite sure, respectively. Generally, the range of scores is 14–70, where scores of 14–32 indicate a low level of self-efficacy, 33–51 moderate self-efficacy, and 52–70 high self-efficacy. This scale has been evaluated psychometrically in the study of Araban et al. and its content and form validities have been confirmed; Cronbach's alpha has also been evaluated to be optimal (0.91) [24].

#### Evaluation of the infants' anthropometric indices

The infant's anthropometric indices will be measured using the ratios of infant weight, height, and head circumference recorded on the infant's health card or health record in the pre-test and post-test stages.

### Data analysis

To describe the state of the data, statistics such as the mean and standard deviation of the main research variables (growth rates of infants, breastfeeding self-efficacy, and quality of life) will be determined, and finally, by using SPSS version 22 the mean score of the main variables of the research before and after the intervention will be compared (if the data are normal) with paired t-test and (if the data are not normal) with the non-parametric Wilcoxon test. The randomized trial, will be the statistical analysis "per protocol". 95% confidence interval and P-value < 0.05 will be considered to investigate the effect of the intervention on the mean of the main research variables.

### Outcome measures

#### Breastfeeding self efficacy of mothers

The mean score of Breastfeeding Self efficacy of mothers will be measured using a questionnaire BSES-SF Dennis in the pre-test and post-test phases.

#### Quality of life of mothers

The mean score of the Quality of Life of mothers will be measured using a questionnaire SF36 in the pre-test and post-test phases.

#### Infants’ growth status

Infant's anthropometric indices (weight-for-age evaluation, height-for-age evaluation, assess weight for height, and assess head circumference for age and body mass index (BMI) for age( will be measured using the ratios of infant weight, height, and head circumference recorded on the infant's health card or health record in the pre-test and post-test stages.

### Ethical considerations

Ethical approval for this study has been obtained by the ethics committee affiliated with Shahid Sadoughi University of Medical Sciences, Yazd, Iran (23/8/2021) reference number IR.SSU.SPH.REC.1400.096), in compliance with the Helsinki Declaration. Ethical considerations include explaining the research objectives to the participants, optional participation in this project, completing the informed consent form by the participants and confidentiality of all information related to the individual and institutional of the study in reporting participation.

## Discussion

The present study will be provided useful protocol about the effect of an educational support intervention for infant care and the sharing of peer group experiences on infants' growth rates, breastfeeding self-efficacy, and quality of life of their mothers.

In other studies conducted in this direction, Masayo Awano et al. [25], Mirghfourvand et al. [26], Azimi and Nasiri [27], Heidari et al. [28], Almohanna et al. [29], Rezaian et al. [30]. Araban et al. [24] showed that educational interventions in the field of infant care and breastfeeding are effective in increasing the quality of life and breastfeeding self-efficacy. Gonzales et al. argued that breastfeeding self-efficacy is a substantially significant variable in influencing breastfeeding outcomes. Breastfeeding self-efficacy in the early postpartum period is an important predictor of breastfeeding duration [31]. Educational intervention programs in the field of women's health can be effective [32, 33].

It is hoped that group training and counseling of mothers and sharing their experiences will increase exclusive breastfeeding and increase mothers' awareness. Increasing the knowledge and attitude of mothers, skills in correct breastfeeding techniques and solving breastfeeding problems in many cultures, including Iranian culture, will be possible with the support of the people around [34].

One of the strengths of this study will be to increase the self-confidence and self-efficacy of mothers to promote breastfeeding. Peer training will be a significant positive point of this study. In this method, the active participation of mothers and cooperation with health care workers will help to promote growth, knowledge and experimental skills. Sharing mothers' experiences and receiving group training will strengthen the enthusiasm and sense of empathy in mothers, and mothers will realize that breastfeeding problems exist for all mothers, so they will be motivated to promote breastfeeding. Another strength of this study will be the language, culture and social characteristics shared by mothers. Breastfeeding self-efficacy is a modifiable and potential factor that will play an effective role in promoting breastfeeding. Also, Breastfeeding self-efficacy will be a suitable theoretical framework to guide effective interventions in improving children's health.

Generalizing of the results of the study according to the cultural, social and nutritional differences in different societies will be one of the challenges of this study. The non-generalization of the study results to mothers of premature infants and mothers with twin infants or mothers and infants with special conditions will be a restriction. The limitation of the statistical population, the busyness of mothers, neglect of educational packages, receiving information by mothers from other members of the health team, and sharing information and experiences of mothers outside the group and without the supervision of researchers will be other restrictions of this study. This study may not be generalizable because people without telephones will be excluded from participating in the intervention, and this is another limitation of this study.

However, According to the investigations, the current educational package not only combines virtual education and peer experiences in strengthening the education of mothers, but also it can improve mothers' physical and mental health and reduce medical costs by using telephone follow-up in supporting the mothers of infants. Additionally, the strategy used in this protocol can be used for education and support in pre-pregnancy, pregnancy and childbirth care. The strategies of this program could be important and cost-effective, and therefore we hope that the success of such a program is a step forward in improving maternal and infant health status.

## Data Availability

Not applicable.

## Abbreviations

QOF: Quality of life
BSES-SF: Breastfeeding self-efficacy
BMI: Body mass index
WHO: World Health Organization
