# Correction: The effect of educational support intervention including peer groups for infant care on the growth rates of infants, breastfeeding self-efficacy and quality of life of their mothers in Iran: study protocol

**DOI:** 10.1186/s12978-022-01555-y

**Published:** 2023-01-12

**Authors:** Forogh Okhovat, Shirin Okhovat, Zohreh Karimiankakolaki, Nooshin Yoshany

**Affiliations:** 1 Nursing and Midwifery Care Research Center, School of Nursing and Midwifery, Iran University of Medical Science, Tehran, Iran; 2 Student Research Committee, School of Nursing and Midwifery, Isfahan University of Medical Sciences, Isfahan, Iran; 3 Department of Health, Shahrekord Branch, Islamic Azad University, Shahrekord, Iran; 4 Department of Health Education and Health Promotion, Social Determinants of Health Research Center, School of Public Health, Shahid Sadoughi University of Medical Sciences, Yazd, Iran

**Correction: Reproductive Health (2022) 19:214** **https://doi.org/10.1186/s12978-022-01523-6**

After publication of this article [1], the authors reported that the author name ‘Nooshin Yoshany’ was incorrectly written as ‘Nooshin Yoshani’.

The original article [1] has been corrected.
